# Two Polarity-Sensitive Fluorescent Probes Based on Curcumin Analogs for Visualizing Polarity Changes in Lipid Droplets

**DOI:** 10.3390/molecules28186626

**Published:** 2023-09-14

**Authors:** Lin Shan, Xuewei Li, Xiuli Zheng, Jiasheng Wu, Haohui Ren, Weimin Liu, Pengfei Wang

**Affiliations:** 1 Key Laboratory of Photochemical Conversion and Optoelectronic Materials and CityU-CAS Joint Laboratory of Functional Materials and Devices, Technical Institute of Physics and Chemistry, Chinese Academy of Sciences, Beijing 100190, China; 2 School of Future Technology, University of Chinese Academy of Sciences, Beijing 100049, China; 3 Institute of Optical Physics and Engineering Technology, Qilu Zhongke, Jinan 250000, China

**Keywords:** polarity-sensitive, lipid droplet targeted, fluorescent probes, cell imaging

## Abstract

As a class of highly dynamic organelles, lipid droplets (LDs) are involved in numerous physiological functions, and the changes in polarity of LDs are closely related to a variety of diseases. In this work, we developed two polarity-sensitive fluorescent probes (CC-CH and CC-Cl) based on curcumin analogs. CC-CH and CC-Cl with a donor–acceptor–donor (D–A–D) structure exhibited the property of intramolecular charge transfer (ICT); thus, their fluorescence emissions were significantly attenuated with increasing ambient polarity. Cell experiments indicated that CC-CH and CC-Cl showed excellent photostability, a low cytotoxicity, and a superior targeting ability regarding LDs. After treatment with oleic acid (OA) and methyl-β-cyclodextrin (M-β-CD), the polarity changes of LDs in living cells could be visualized by using CC-CH and CC-Cl. In addition, CC-CH and CC-Cl could monitor polarity changes of LDs in different pathological processes, including inflammatory responses, nutrient deprivation, and H_2_O_2_-induced oxidative stress. Therefore, CC-CH and CC-Cl are promising potential fluorescent probes for tracking intracellular LD polarity changes.

## 1. Introduction

Lipid droplets (LDs, also known as lipid bodies or oil bodies) play an indispensable role in the maintenance of cellular energy homeostasis as the center of intracellular lipid storage, metabolism, and transport [1,2,3]. LDs have neutral lipids [4,5] (the most common components in eukaryotes are triacylglycerols and sterol esters) as the center, surrounded by a phospholipid monolayer modified with a variety of associated proteins [6,7]. They are not an inert organelle, as recognized by humans in earlier times, but are highly dynamic and tightly connected to various organelles [8,9,10]. LDs are involved in numerous physiological functions including signaling [11,12], buffering of lipotoxicity [13,14], relief of cellular stress [15,16,17], and modulation of inflammatory responses [18,19]. Dysregulation of LDs is associated with diseases such as obesity [20,21], cardiovascular diseases [22,23], non-alcoholic fatty liver disease (NAFLD) [24,25,26], diabetes [27,28,29], neurodegeneration [30,31], and multiple cancers [32,33]. Dysregulation of LDs is usually accompanied by changes in the internal environment of the LDs. Polarity, on the other hand, as an important parameter in the internal environment of LDs, has been proven to be associated with a variety of disease processes [34,35]. Consequently, the development of tools to visualize changes in the polarity of LDs is of great importance for the early diagnosis of diseases and the exploration of disease processes.

Due to the advantages of a high spatial resolution, high sensitivity, non-invasive nature, simple operation, and low cost, fluorescence imaging technology has a wide range of applications in life sciences, medical diagnosis, and drug development [36,37]. In recent years, with increasing awareness of the importance of LDs, LD-targeted fluorescent probes based on various fluorescent parent nuclei have been developed [38,39,40]. However, there are still relatively few probes capable of imaging changes in the polarity of LDs [41], and the photostability of most of them needs to be further improved. Furthermore, the majority of polarity-sensitive LD probes have not yet delved into the relationship between LD polarity and associated pathological processes.

The intramolecular charge transfer (ICT) mechanism [42] has been widely adopted in the construction of LD-targeted polarity-sensitive probes due to the advantages of easy realization and easy adjustment. Polarity-sensitive probes based on the ICT mechanism are usually realized by constructing a π-bonded D–A system [43], where D and A represent an electron donor and an electron acceptor, respectively. In low polarity solvents, the excited state of the probe is presented in the locally excited (LE) state, leading to strong fluorescence emission at short wavelengths, whereas in high polarity solvents, due to strong dipole–dipole interactions between the solvent and the probe, the excited state of the probe exists in a lower energy ICT state, leading to weak fluorescence emission at long wavelengths.

In recent years, a few fluorescent probes based on the D–A structure have been developed for the polarity detection of intracellular LDs [44,45,46,47]. For instance, Lin et al. [44] reported a D–A molecule with ICT properties, CTPA, which was sensitive to polarity and specifically targeted LDs. CTPA could distinguish between cancer cells and normal cells based on the difference in polarity. Klymchenko et al. [45] developed a fluorescent probe with the D–A structure: Dimethyl Aniline Furaldehyde (DAF). Under a single excitation source (405 nm laser), DAF enabled the detection of polarity differences within LDs of the same cell, revealing polarity heterogeneity from the core to the edge. Compared with conventional D–A molecules, D–A–D molecules have dual ICT processes due to the fact that the electron acceptor is connected to two electron donors at the same time [48]. They are more favorable for the charge separation of the probe in high polarity solvents as well as the lowering of the excited state energy level, resulting in a higher polarity sensitivity. However, D–A–D molecules for detecting the polarity of LDs have rarely been reported.

**Scheme 1:**
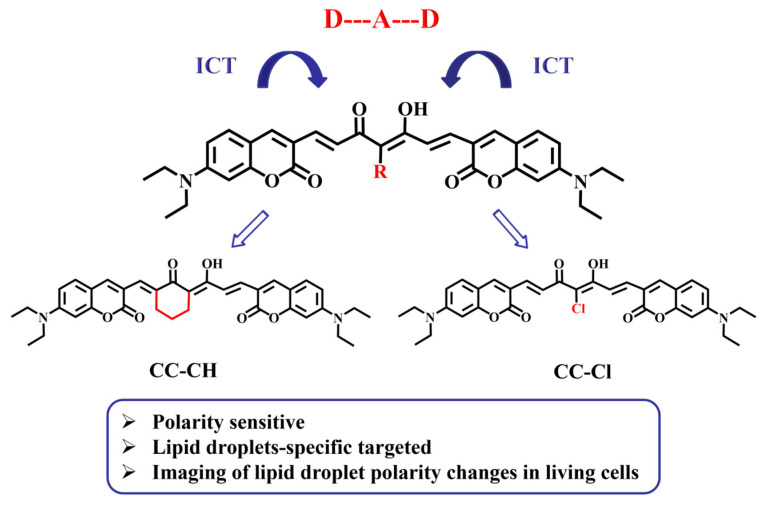
Structure of CC-CH and CC-Cl.

In our previous work, we designed an LD-targeted photosensitizer with a D–A–D structure by introducing coumarin into the curcumin skeleton, which exhibited clear photocytotoxicity in a hypoxic microenvironment [49]. In this work, we synthesized two new D–A–D fluorescent probes (CC-CH and CC-Cl) based on curcumin analogs with excellent LD-targeting abilities. In order to increase the stability and extend the emission wavelength, a six membered ring and a chlorine atom were introduced into the coumarin–curcumin framework, respectively (Scheme 1), forming two D−A−D molecules with ICT properties. CC-CH and CC-Cl are polarity sensitive, emit strong fluorescence in low polarity solvents, and significantly attenuate fluorescence in high polarity solvents due to the dipole−dipole interactions in high polarity solvents. Utilizing the superior targeting ability to LDs and good photostability, CC-CH and CC-Cl allow imaging of changes in the size and number of LDs. More significantly, these polarity-sensitive probes can be successfully used for visualization of LD polarity changes in different pathological processes, such as inflammatory responses, nutrient deprivation, and H_2_O_2_-induced oxidative stress.

## 2. Results and Discussion

### 2.1. Photophysical Properties

**Figure 1:**
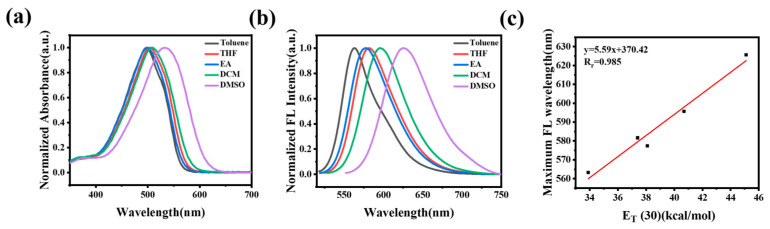
(**a**) Normalized absorption spectra of CC-CH in different polarity solvents. (**b**) Normalized fluorescence spectra of CC-CH in different polarity solvents. (**c**) Plot of the maximum emission wavelength of CC-CH in different polarity solvents vs. E_T_ (30).

Initially, we investigated the photophysical properties of CC-CH and CC-Cl in organic solvents with different polarities. As shown in Figure 1a, the absorption spectra of CC-CH showed a positive solvatochromic shift from toluene (498 nm) to DMSO (532 nm), which is attributed to the ICT effect. Correspondingly, the fluorescence spectra of CC-CH were red-shifted from 563 nm to 626 nm with increasing solvent polarity (Figure 1b), suggesting that CC-CH is sensitive to polarity. To further explore the relationship between fluorescence emission and solvent polarity, we chose the solvent polarity parameter E_T_(30) [50] for solute–solvent interactions at the microscopic level as a measure of the magnitude of solvent polarity. Typically, solvent polarity shows a positive correlation with E_T_(30). As shown in Figure 1c, the maximum emission wavelength of CC-CH in different polarity solvents had a good linear relationship with E_T_(30) of the solvents after fitting calculations (y = 5.59x + 370.42, R_r_ = 0.985). Other than this, the molar extinction coefficients of CC-CH in different polarity solvents were all quite high (Table S1), and were all around 80,000 M^−1^cm^−1^. This shows excellent light absorption ability, which is undoubtedly beneficial for fluorescence imaging. The fluorescence quantum yields of CC-CH in different solvents were 12.2% (toluene), 12.5% (THF), 11.7% (EA), and 13.5% (DCM) (Table S1). In contrast, CC-CH showed weak fluorescence emission in DMSO with a fluorescence quantum yield of only 3.3%.

**Figure 2:**
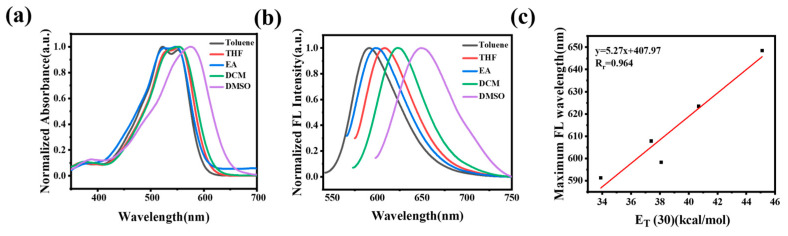
(**a**) Normalized absorption spectra of CC-Cl in different polarity solvents. (**b**) Normalized fluorescence spectra of CC-Cl in different polarity solvents. (**c**) Plot of the maximum emission wavelength of CC-Cl in different polarity solvents vs. E_T_ (30).

Similar to CC-CH, a positive solvatochromism of CC-Cl and a good linear relationship between the maximum emission wavelength of CC-Cl and E_T_(30) of the solvents were also obtained (Figure 2). However, the maximum absorption and emission wavelengths of CC-Cl were both red-shifted by about 30 nm compared with CC-CH (Table S2), which is attributed to the introduction of the electron-withdrawing chlorine atom. In addition, CC-Cl had lower molar extinction coefficients and higher fluorescence quantum yields than CC-CH in different solvents (Table S2).

**Figure 3:**
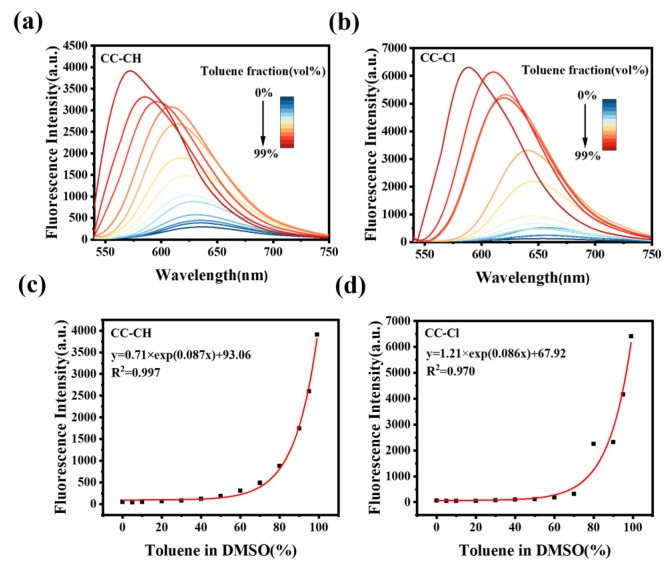
Fluorescence spectra of (**a**) CC-CH and (**b**) CC-Cl in DMSO/toluene mixtures with different toluene fractions. Plots of fluorescence intensity of (**c**) CC-CH and (**d**) CC-Cl in DMSO/toluene mixtures vs. different toluene fractions. CC-CH: λ_ex_ = 498 nm, CC-Cl: λ_ex_ = 519 nm.

In order to accurately evaluate the fluorescence intensity versus polarity, the fluorescence spectra of CC-CH and CC-Cl were measured in DMSO/toluene mixtures. As shown in Figure 3a, when increasing the proportion of toluene, the fluorescence spectra of CC-CH were blue-shifted and the corresponding fluorescence intensity was significantly enhanced. When the proportion of toluene was increased from 0% to 99%, the fluorescence intensity of CC-CH increased to about 76 times that of the original one. After nonlinear fitting calculations, there was a certain pattern between the fluorescence intensity of CC-CH and the toluene ratio, namely, the fluorescence intensity grew exponentially with increasing the toluene ratio (Figure 3c). The change in fluorescence emission of CC-Cl in DMSO/toluene mixtures was similar to CC-CH (Figure 3b), but the increase in fluorescence intensity was greater, becoming 103 times more than the original (Figure 3d). This suggests that CC-Cl may be more sensitive to the decrease in environmental polarity in comparison with CC-CH.

Considering the diversity and complexity within cells, we also measured the fluorescence spectra of CC-CH and CC-Cl in H_2_O/1,4-dioxane mixtures (Figure S1a,b). Similar to the results in the DMSO/toluene mixtures, when the proportion of 1,4-dioxane was increased from 0% to 99%, the fluorescence intensities of CC-CH and CC-Cl rose by approximately 180 and 457 times, respectively (Figure S1c,d). The increase in fluorescence intensity of CC-Cl was also higher than CC-CH in the H_2_O/1,4-dioxane mixtures, further verifying that CC-Cl is more sensitive to low polarity environments than CC-CH. All of these results indicate that CC-CH and CC-Cl have polarity-sensitive properties and may be used as polarity-sensitive fluorescent probes.

### 2.2. LD-Specific Ability

At first, cytotoxicity assays were performed in three tumor cell lines (HepG-2, HeLa, and A549) and one normal cell line (HU-EVC) to assess the biocompatibility of CC-CH and CC-Cl as probes. The MTT results showed that the probes at 0–5 μM concentrations had low cytotoxicity after 24 h of co-incubation with the four cell lines mentioned above, and the cell viability was greater than 80% (Figure S2). The cytotoxicity of CC-CH was slightly higher than CC-Cl, probably due to the introduction of the six membered ring, which increases the cytotoxicity of CC-CH.

In view of the diversity and complexity of the intracellular environment, anti-interference is particularly important for fluorescent probes. To comprehensively assess the effect of the intracellular environment on CC-CH and CC-Cl, we tested a variety of potential interferences, including pH, anions and cations, hydrogen peroxide (H_2_O_2_), and amino acids. As shown in Figure S3, the absorption spectra of CC-CH and CC-Cl showed little change in the pH 4–9 range, showing good pH stability. Moreover, the fluorescence intensity of CC-CH and CC-Cl changed less after the addition of various anions and cations, H_2_O_2_, and amino acids, indicating that CC-CH and CC-Cl have favorable anti-interference properties (Figure S4). The photostability testing of the probes and the commercial LD probe BODIPY 493/503 in HepG-2 cells showed that CC-CH and CC-Cl possessed more superior photostabilities than BODIPY 493/503 (Figure S5), and the fluorescence intensity remained more than 85% of the original fluorescence intensity after 10 min of continuous scanning with a confocal laser scanning microscope (CLSM). All of the above tests confirm that CC-CH and CC-Cl have various properties that fluorescent probes should have for cellular imaging.

**Figure 4:**
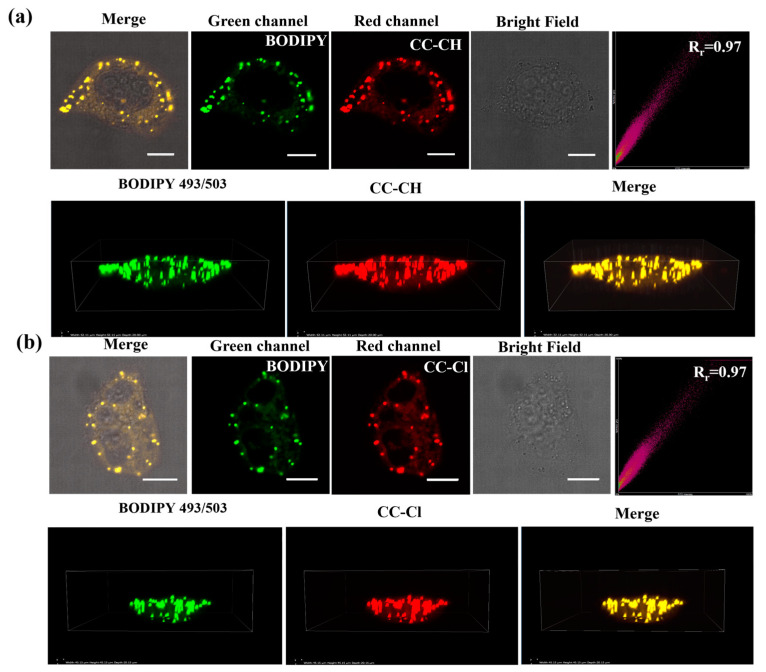
Colocalization images and three-dimensional images of (**a**) CC-CH (2.5 μM) and (**b**) CC-Cl (2.5 μM) with BODIPY 493/503 (0.2 μM) in HepG-2 cells. Scale bar: 10 μm. Green channel: λ_ex_ = 488 nm, λ_em_ = 500–530 nm. Red channel: λ_ex_ = 488 nm, λ_em_ = 568–643 nm.

To demonstrate that the probes could enter the cells and target the LDs, confocal fluorescence images of CC-CH and CC-Cl incubated with HepG-2 cells for different durations were firstly taken. CC-CH and CC-Cl already exhibited good fluorescence imaging after 1 h of co-incubation with HepG-2 cells at a low concentration (Figure S6). To further validate the specific targeting ability of CC-CH and CC-Cl to LDs, co-localization experiments with three commercial green-emitting organelle fluorescent probes were performed. As shown in Figure 4, CC-CH and CC-Cl achieved a high Pearson correlation coefficient (R_r_) of 0.97 with the commercial LD probe BODIPY 493/503, as well as overlapping almost completely at a three-dimensional level. However, CC-CH and CC-Cl specifically targeted LDs while overlapping poorly with the lysosomal probe Lyso Tracker Green (LTG) and the mitochondrial probe Mito Tracker Green (MTG) (Figures S7 and S8). The anti-light interference testing also indicated that the Pearson correlation coefficient with commercial LD probe BODIPY 493/503 had a high confidence (Figures S9 and S10). In addition to HepG-2 cells, co-localization experiments with BODIPY 493/503 were also performed in Hela cells and A549 cells (Figures S11 and S12), and the Pearson correlation coefficients were all above 0.95, indicating that CC-CH and CC-Cl have excellent LD targeting abilities in different cell lines.

### 2.3. Fluorescence Imaging of LDs Polarity Changes

**Figure 5:**
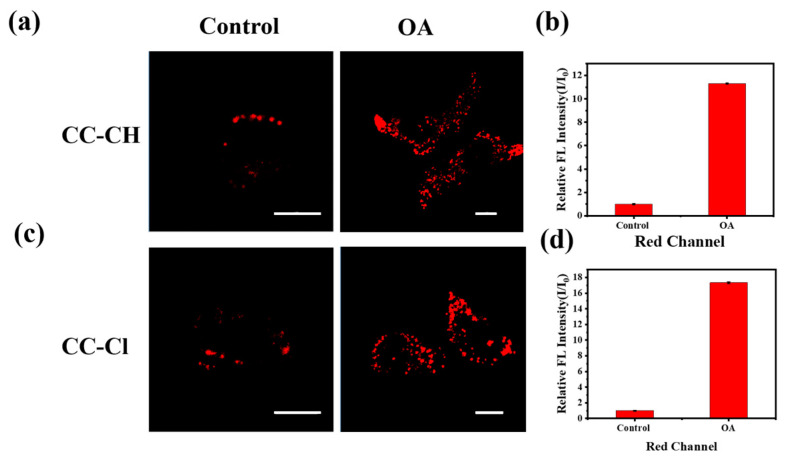
Confocal fluorescence images of HepG-2 cells treated with 0.1 mM OA for 4 h and then stained with (**a**) CC-CH and (**c**) CC-Cl. The relative fluorescence intensity (I/I_0_) of (**b**) CC-CH and (**d**) CC-Cl of the control and OA-treated groups in the red channel. Scale bar: 10 μm. Red channel: λ_ex_ = 488 nm, λ_em_ = 568–643 nm.

To verify whether the probe could image changes in the polarity of intracellular LDs, HepG-2 cells were treated with oleic acid (OA) for 4 h, and then washed off and co-incubated with the probe. OA, a high nutrient that is extremely easily absorbed by cells, can decrease the polarity of intracellular LDs and promote the production of large amounts of endogenous LDs [51]. As shown in Figure 5a,c, the fluorescence in LDs markedly increased after treatment with OA compared to the untreated group, suggesting that the probe can be used to visualize changes in the polarity of intracellular LDs. Furthermore, there was a significant boost in the number and size of LDs after treatment with OA, suggesting that the probes can not only reveal changes in the polarity of intracellular LDs but also changes in the size and number of intracellular LDs. Quantitative analysis of the fold increase in the average intracellular fluorescence intensity revealed that the fluorescence increase of CC-Cl was more significant than CC-CH (Figure 5b,d). This result further demonstrates at the cellular imaging level that CC-Cl is more sensitive to a decrease in LD polarity compared with CC-CH.

Methyl-β-cyclodextrin (M-β-CD), a recognized biomembrane cholesterol scavenger [52], is able to destroy the LD microenvironment, thereby increasing the polarity of LDs. To further verify the ability of the probe to indicate intracellular LD polarity changes, HepG-2 cells treated with M-β-CD were stained with the probe (Figure S13). Compared with the control group, the fluorescence intensity of LDs in HepG-2 cells treated with M-β-CD was somewhat attenuated, which successfully indicated the increase in the polarity in LDs. Both of these results demonstrate that CC-CH and CC-Cl can be used as indicators of the change in polarity of intracellular LDs.

### 2.4. Monitoring of LD Polarity Changes during Different Pathological Processes

**Figure 6:**
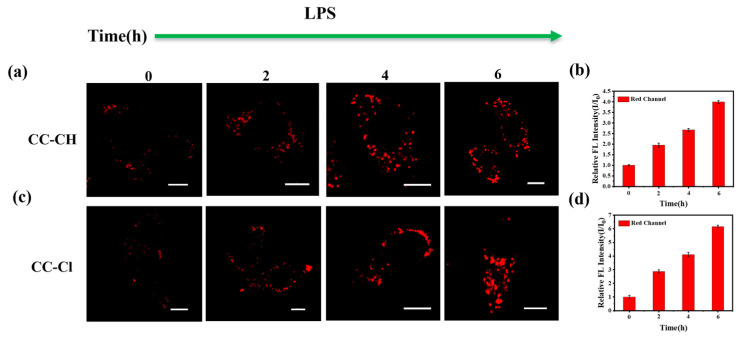
Confocal fluorescence images of HepG-2 cells treated with LPS for different times of 0, 2, 4, and 6 h and stained with (**a**) CC-CH and (**c**) CC-Cl. The relative fluorescence intensity (I/I_0_) of (**b**) CC-CH and (**d**) CC-Cl at different LPS treatment times. Scale bar: 10 μm. Red channel: λ_ex_ = 488 nm, λ_em_ = 568–643 nm.

As an important class of cellular injury models, inflammatory cells are crucial in medical research [53]. Lipopolysaccharide (LPS), as a major component of Gram-negative bacterial cell walls, is commonly used to obtain inflammatory cell models [54]. Therefore, we used LPS to induce an inflammatory cell model to explore the polarity changes of LDs in the inflammatory cell model. As shown in Figure 6, the fluorescence intensity gradually increased with the extension of LPS induction time, indicating that the polarity of LDs gradually decreases in the inflammatory cell model, which may be related to the accumulation of triglycerides within the LDs under the inflammatory state [55].

**Figure 7:**
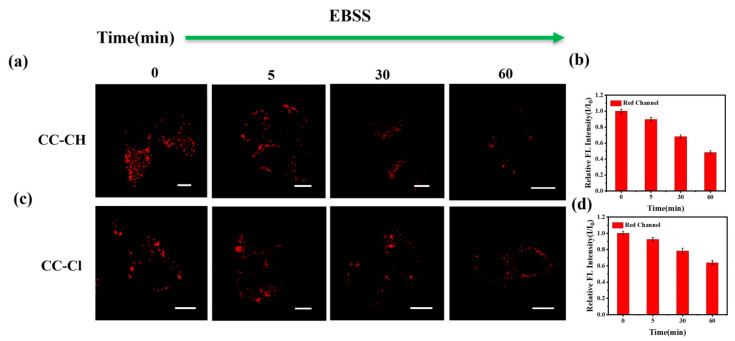
Confocal fluorescence images of HepG-2 cells stained with (**a**) CC-CH (2.5 μM) and (**c**) CC-Cl (2.5 μM) for 1 h and then incubated in EBSS for different times (0, 5, 30, 60 min). The relative fluorescence intensity (I/I_0_) of (**b**) CC-CH and (**d**) CC-Cl at different starvation times. Scale bar: 10 μm. Red channel: λ_ex_ = 488 nm, λ_em_ = 568–643 nm.

Under starvation conditions, LDs release stored fatty acids (FAs) [56], which are available for mitochondrial catabolism to maintain normal cellular functions, and this process is accompanied by an increase in the polarity of LDs. To verify the phenomenon of increased polarity within LDs during this process, we used serum-free Earle’s Balanced Salt Solution (EBSS) for nutrient deprivation of HepG-2 cells after the probes were pre-incubated. As shown in Figure 7, the fluorescence intensity gradually decreased with the prolongation of nutritional deprivation time, indicating a gradual increase in the polarity of LDs under starvation conditions.

**Figure 8:**
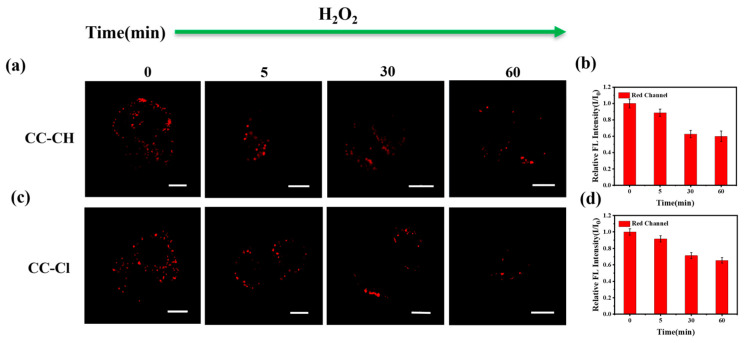
Confocal fluorescence images of HepG-2 cells stained with (**a**) CC-CH (2.5 μM) and (**c**) CC-Cl (2.5 μM) for 1 h and then incubated with 1 mM H_2_O_2_ for different time (0, 5, 30, 60 min). The relative fluorescence intensity (I/I_0_) of (**b**) CC-CH and (**d**) CC-Cl at different H_2_O_2_ stimulation times. Scale bar: 10 μm. Red channel: λ_ex_ = 488 nm, λ_em_ = 568–643 nm.

The oxidative stress state of cells has been shown to be closely related to numerous physiological and pathological processes [57]. It has been reported that high concentrations of H_2_O_2_ can stimulate cells to enter a state of oxidative stress within a short period of time [58]. As shown in Figure 8, the fluorescence in the LDs gradually diminished with the extension of H_2_O_2_ addition time, indicating an increase in the polarity in LDs, which may be due to lipid peroxidation in the oxidative stress state [59]. The above result indicates that CC-CH and CC-Cl are capable of monitoring H_2_O_2_-induced polarity changes in intracellular LDs under oxidative stress.

## 3. Materials and Methods

### 3.1. Materials and Instruments

All reagents were purchased from commercial sources (Innochem (Beijing, China), TCI (Shanghai, China), Sigma-Aldrich (Shanghai, China), Adamas-beta (Shanghai, China), Aladdin (Shanghai, China)) and used without further purification. All solvents and inorganic salts were purchased from Beijing Reagent, Sinopharm Chemical Reagent Beijing or General-Reagent, and used without further processing. Human cervical carcinoma cells (HeLa cells), human hepatocarcinoma cells (HepG-2 cells), human non-small cell lung cancer cell (A549 cells), and Human Umbilical Vein Endothelial Cells (HU-EVC cells) were purchased from the Center of Cells, Peking Union Medical College.

^1^H NMR and ^13^C NMR spectra were obtained with Bruker Avance III 400 and Ascend 600 MHz spectrometers (Karlsruhe, Germany), respectively, using CDCl_3_ or DMSO-d_6_ as a solvent, and tetramethylsilane (TMS) was used as an internal standard. Electrospray Ionization Mass Spectrometry (ESI-MS) was conducted using a Thermo Fisher Scientific Q-Exactive spectrometer (Waltham, MA, USA). Absorption spectra were recorded on a Hitachi U-3900 ultraviolet–visible (UV-Vis) spectrophotometer (Tokyo, Japan). Fluorescence spectra were recorded on a Hitachi F-4600 spectrofluorometer (Tokyo, Japan). Absolute fluorescence quantum yields were measured in a Quantaurus-QY absolute PL quantum yield spectrometer C11347 (Hamamatsu, Shizuoka, Japan).

### 3.2. Synthesis and Characterization

The synthetic route to the CC-CH and CC-Cl probes is shown in Scheme S1. Their characterization (Figures S14–S18) and synthesis details of the intermediates [60] are provided in the Electronic Supplementary Information.

*7-(diethylamino)-3-((E)-((Z)-3-((E)-3-(7-(diethylamino)-2-oxo-2H-chromen-3-yl)-hydroxyallylidene)-2-oxocyclohexylidene) methyl)-2H-chromen-2-one (CC-CH)*: Boron trioxide (1.05 g, 15 mmol) was added to a two-necked flask and then 8 mL anhydrous DMF was added under a nitrogen atmosphere. The temperature was raised to 120 °C and the mixture was stirred for 5 min. Then, 2-acetylcyclohexanone (133 mmL, 1 mmol), tributyl borate (0.54 mL, 2 mmol), compound 2 (522 mg, 2.1 mmol), and 1, 2, 3, 4-tetrahydroquinoline (0.1 mL) were successively added, and the resulting mixture was stirred at 120 °C for 5 h. The mixture was extracted with dichloromethane (DCM) 3 times. The combined organic layers were washed with ultrapure water 6 times and dried over MgSO_4_. Finally, a red solid (CC-CH) (289 mg, 48%) was obtained by silica gel column purification. ^1^H NMR (400 MHz, Chloroform-*d*) δ 17.03 (s, 1H), 7.77 (d, *J* = 15.2 Hz, 1H), 7.71 (s, 2H), 7.60–7.50 (m, 2H), 7.34–7.28 (m, 2H), 6.61 (d, *J* = 9.0 Hz, 2H), 6.50 (s, 2H), 3.49–3.39(m,8H), 2.70 (t, *J* = 6.1 Hz, 4H), 1.81 (t, *J* = 6.2 Hz, 2H), 1.29–1.18 (m,12H).^13^C NMR (151 MHz, CDCl_3_-*d*) δ 186.54, 176.76, 161.83, 160.23, 156.40, 156.08, 151.71, 150.90, 145.47, 141.75, 137.37, 134.23, 129.97, 129.46, 126.66, 122.21, 116.90, 115.29, 109.51, 109.39, 109.14, 109.02, 108.63, 97.18, 96.83, 45.03, 44.92, 27.79, 24.23, 22.83, 12.50. HRMS (ESI-MS, *m*/*z*): [M+H]^+^ calcd for C_36_H_39_N_2_O_6_, 595.2803; Found, 595.2801.
*3,3′-((1E,3E,6E)-4-chloro-3-hydroxy-5-oxohepta-1,3,6-triene-1,7-diyl)bis(7(diethylamino)-2H-chromen-2-one) (CC-Cl)*: Boron trioxide (1.05 g, 15 mmol) was added to a two-necked flask and then 8 mL anhydrous DMF was added under a nitrogen atmosphere. The temperature was raised to 120 °C and the mixture was stirred for 5 min. Then, 3-Chloroacetylacetone (118 mmL, 1 mmol), tributyl borate (0.54 mL, 2 mmol), compound 2 (522 mg, 2.1 mmol), and 1, 2, 3, 4-tetrahydroquinoline (0.1 mL) were successively added, and the resulting mixture was stirred at 120 °C for 5 h. The mixture was extracted with dichloromethane (DCM) 3 times. The combined organic layers were washed with ultrapure water 6 times and dried over MgSO_4_. Finally, a red solid (CC-Cl) (332 mg, 56%) was obtained by silica gel column purification. ^1^H NMR (400 MHz, CDCl_3_-*d*) δ 16.55 (s, 1H), 7.90 (d, *J* = 15.5 Hz, 2H), 7.79 (s, 2H), 7.64 (d, *J* = 15.5 Hz, 2H), 7.33 (d, *J* = 8.9 Hz, 2H), 6.61 (d, *J* = 9.0 Hz, 2H), 6.51 (s, 2H), 3.45 (q, *J* = 7.0 Hz, 8H), 1.26–1.22 (m, 12H).^13^C NMR (Not obtained due to insufficient solubility).HRMS (ESI-MS, *m*/*z*): [M+H]^+^ calcd for C_33_H_34_ClN_2_O_6_, 589.2100; Found, 589.2101.

### 3.3. Photophysical Properties Measurements

CC-CH and CC-Cl were dissolved in DMSO to prepare a 1.0 mM stock solution. Afterwards, the stock solution was stored at −20 °C for future use. The stock solution of CC-CH was diluted to 10 μM with various solvents to measure the absorption and fluorescence spectra. The stock solution of CC-Cl was diluted to 20 μM with various solvents to measure the absorption and fluorescence spectra. The excitation wavelength corresponded to the maximum absorption wavelength. The fluorescence spectra of CC-CH and CC-Cl were also measured in DMSO/toluene mixtures and H_2_O/1,4-dioxane mixtures, respectively, to confirm the established relationship between the fluorescence of CC-CH and CC-Cl and the polarity of solvent mixtures. The fluorescence spectra of the DMSO/toluene system were measured using the maximum absorption wavelength of the probe in toluene as the excitation wavelength under identical conditions. Similarly, the fluorescence spectra of the H_2_O/1,4-dioxane system were measured with the maximum absorption wavelength of the probe in 1,4-dioxane as the excitation wavelength.

The absorption spectra of CC-CH and CC-Cl in phosphate-buffered saline (PBS) with different pH values (pH 4–9, 1%DMSO) were measured to assess their pH stability. PBS with different pH values was calibrated with a pH meter before use.

The solutions of interfering substances for anti-interference testing were freshly prepared. Anti-interference testing was performed in PBS (pH = 7.4, 1%DMSO). The concentrations of all anions and cations, H_2_O_2_, and amino acids were 100 μM.

### 3.4. Biological Evaluation

#### 3.4.1. Cell Cultures

The HepG-2, HeLa, and HU-EVC cells were cultured in a medium containing 89% Dulbecco’s Modified Eagle Medium (DMEM), 10% FBS, 0.05%, 100 μg/mL penicillin + 0.05%, and 100 μg/mL streptomycin in a Petri dish under a 5% CO_2_ atmosphere at 37 °C for 24 h. The A549 cells were cultured in a medium containing 89% McCoy’s 5A Medium (5A), 10% FBS, 0.05%, 100μg/mL penicillin + 0.05%, and 100 μg/mL streptomycin in a Petri dish under a 5% CO_2_ atmosphere at 37 °C for 24 h.

#### 3.4.2. Cytotoxicity Assay of CC-CH and CC-Cl

The probes cytotoxicity to HeLa, HepG-2, A549, and HU-EVC cells was evaluated by an MTT (3-(4,5-dimethylthiazol-2-yl)-2,5-diphenyltetrazo-lium bromide) assay. Exponentially grown cells were seeded at a density of 5 × 10^4^ cells/mL into 96-well plates and cultured for 24 h. Then, the cells were treated with tested samples at different concentrations (1, 2, 3, 4, and 5 µM) for 24 h. Control cells were treated with cell culture broth alone. After incubation at 37 °C for 24 h, the MTT assay was conducted. The absorbance was measured at 570 nm using a multifunctional microplate reader. The relative cell viability (%) was calculated by the following formula:

cell viability (%) = OD_treated_/OD_control_ × 100.

#### 3.4.3. Cell Imaging at Different Incubation Times and Photostability Testing

HepG-2 cells were incubated with CC-CH (2.5 μM) or CC-Cl (2.5 μM) for different incubation durations (0.5 h, 1 h, 2 h), washed thrice with PBS, and then placed in 1 mL PBS for imaging with CLSM. Fluorescence signals were collected at 568–643 nm using a laser at 488 nm as the excitation source.

HepG-2 cells were incubated with CC-CH/CC-Cl (2.5 μM) and BODIPY 493/503 (0.2 μM) and then irradiated with CLSM for 10 min continuously (scan every two seconds, 300 times in total). The change in fluorescence intensity was recorded. The fluorescence signals were collected at 568–643 nm for CC-CH/CC-Cl and 500–530 nm for BODIPY 493/503 using a laser at 488 nm as the excitation source.

#### 3.4.4. Cellular Co-Localization Experiments

HepG-2, HeLa, and A549 cells were incubated with CC-CH (2.5 μM) or CC-Cl (2.5 μM) for 1 h, washed thrice with PBS, and then incubated with BODIPY 493/503 (0.2 μM), LTG (0.2 μM), or MTG (0.2 μM) for 10 min. Confocal fluorescence images were captured with the green channel (λ_ex_ = 488 nm, λ_em_ = 500–530 nm) for the organelle-targeted fluorescent probe and the red channel (λ_ex_ = 488 nm, λ_em_ = 568−643 nm) for CC-CH and CC-Cl.

#### 3.4.5. Visualization of LD Polarity Changes in HepG-2 Cells

HepG-2 cells were treated with 0.1 mM OA for 4 h and washed thrice with PBS, then the cells were incubated with CC-CH (2.5 μM) or CC-Cl (2.5 μM) for 1 h. In the control group, HepG-2 cells were only incubated with CC-CH (2.5 μM) or CC-Cl (2.5 μM) for 1 h. Fluorescence images were collected with CLSM. Red channel: λ_ex_ = 488 nm, λ_em_ = 568–643 nm.

HepG-2 cells were treated with 2 mg/mL M-β-CD for 2 h, washed thrice with PBS, and then incubated with CC-CH (2.5 μM) or CC-Cl (2.5 μM) for 1 h. In the control group, HepG-2 cells were only incubated with CC-CH (2.5 μM) or CC-Cl (2.5 μM) for 1 h. The fluorescence images were collected with CLSM. Red channel: λ_ex_ = 488 nm, λ_em_ = 568–643 nm.

HepG-2 cells were treated with LPS for different durations of 0, 2, 4, and 6 h and then the cells were incubated with CC-CH (2.5 μM) or CC-Cl (2.5 μM) for 1 h. Fluorescence images were collected with CLSM. Red channel: λ_ex_ = 488 nm, λ_em_ = 568–643 nm.

HepG-2 cells were incubated with CC-CH (2.5 μM) or CC-Cl (2.5 μM) for 1 h, washed thrice with PBS, and then treated with 1 mL EBSS or 1 mM H_2_O_2_ for different durations (0, 5, 30, or 60 min). Fluorescence images were collected with CLSM. Red channel: λ_ex_ = 488 nm, λ_em_ = 568–643 nm.

## 4. Conclusions

In summary, we have constructed two fluorescent probes with the D–A–D structure, CC-CH and CC-Cl, which are sensitive to polarity and exhibit positive solvatochromism due to the ICT property. Cell experiments showed that CC-CH and CC-Cl show low cytotoxicity, good photostability, and specific targeting of LDs. The excellent properties of the two probes in solution and in living cells enable them to be used for monitoring LD polarity changes in different pathological processes, including inflammatory responses, nutrient deprivation, and oxidative stress. CC-CH and CC-Cl present as potential tools for probing changes in LD polarity.

## Data Availability

The data from this study are available from the corresponding author upon reasonable request.

## Supplementary Materials

The following supporting information can be downloaded at: https://www.mdpi.com/article/10.3390/molecules28186626/s1, Table S1: Photophysical properties of CC-CH in different polarity solvents; Table S2: Photophysical properties of CC-Cl in different polarity solvents; Figure S1: Fluorescence changes in CC-CH and CC-Cl in H_2_O/1,4-dioxane mixtures with different 1,4-dioxane fractions; Figure S2: Cytotoxicity testing; Figure S3: pH stability testing; Figure S4: Anti-interference testing; Figure S5: Photostability testing in HepG-2 cells; Figure S6: CLSM images of CC-CH and CC-Cl in HepG-2 cells at different incubation times; Figure S7: Colocalization images of CC-CH with LTG and MTG in HepG-2 cells; Figure S8: Colocalization images of CC-Cl with LTG and MTG in HepG-2 cells; Figure S9: Anti-light interference testing of CC-CH and BODIPY 493/503; Figure S10: Anti-light interference testing of CC-Cl and BODIPY 493/503; Figure S11: Colocalization images of CC-CH with BODIPY 493/503 in different cell lines; Figure S12: Colocalization images of CC-Cl with BODIPY 493/503 in different cell lines; Figure S13: Visualization of the M-β-CD-induced LD polarity increase in living cells; Figures S14–S18: NMR and MS spectra; Scheme S1: Synthetic route to CC-CH and CC-Cl; Synthesis details of the intermediates.

## Sample Availability

Samples of the compounds CC-CH and CC-Cl are available from the authors.
