# Supplementary material for: Two Polarity-Sensitive Fluorescent Probes Based on Curcumin Analogs for Visualizing Polarity Changes in Lipid Droplets
Source: Molecules. 2023 Sep 14;28(18):6626. doi: 10.3390/molecules28186626 (PMC10535065; doi:10.3390/molecules28186626)
Supplement: Supplementary file 1 [file molecules-28-06626-s001.zip › molecules-2606166-supplementary.pdf]

Electronic Supplementary Information (ESI) for

Two polarity-sensitive fluorescent probes based on curcumin analogs for visualizing polarity changes in lipid droplets

Lin Shan, Xue-Wei Li, Xiu-Li Zheng, Jia-Sheng Wu, Hao-Hui Ren, Wei-Min Liu*, and Peng-Fei Wang

Table of Contents

**Section**	**Title**	**Pages**
Table S1	Photophysical properties of CC-CH in different polarity solvents	S2
Table S2	Photophysical properties of CC-Cl in different polarity solvents	S2
Figure S1	Fluorescence changes of CC-CH and CC-Cl in H_2O/1,4-dioxane<br>mixtures with different 1,4-dioxane fractions.	S3
Figure S2	Cytotoxicity testing	S3
Figure S3	pH stability testing	S4
Figure S4	Anti-interference testing	S4
Figure S5	Photostability testing in HepG-2 cells.	S5
Figure S6	CLSM images of CC-CH and CC-Cl in HepG-2 cells at different incubation times.	S5
Figure S7	Colocalization images of CC-CH with LTG and MTG in HepG-2 cells.	S6
Figure S8	Colocalization images of CC-Cl with LTG and MTG in HepG-2 cells.	S6
Figure S9	Anti-light interference testing of CC-CH and BODIPY 493/503	S7
Figure S10	Anti-light interference testing of CC-Cl and BODIPY 493/503	S7
Figure S11	Colocalization images of CC-CH with BODIPY 493/503 in different cell lines.	S8

Figure S12	Colocalization images of CC-Cl with BODIPY 493/503 in different cell lines.	S8
Figure S13	Visualization of M-β-CD-induced LD polarity increase in HepG-2 cells	S9
Scheme S1	Synthetic route to CC-CH and CC-Cl	S9
Methods	Synthesis details of the intermediates	S10
Figures S14-18	NMR and MS spectra	S11-13
References		S13

Table S1. Photophysical properties of CC-CH in different polarity solvents.

solvent	$E_T(30)^a$ (kcalmol^{-1})	λ_{abs} (nm)	λ_F (nm)	stokes shift (cm^{-1})	ε (M^{-1}cm^{-1})	Φ_F ^{b} (%)
**Toluene**	33.9	498	563	2318	99300	12.2
**THF**	37.4	504	582	2659	85300	12.5
**EA**	38.1	499	578	2739	75400	11.7
**DCM**	40.7	508	596	2907	94600	13.5
**DMSO**	45.1	532	626	2823	72000	3.3

^{a}**E_T(30)** is the solvent polarity parameter[1].

^{b}**Φ_F** is the absolute fluorescence quantum yield with an integrating sphere.

Table S2. Photophysical properties of CC-Cl in different polarity solvents.

solvent	$E_T(30)^a$ (kcalmol^{-1})	λ_{abs} (nm)	λ_F (nm)	stokes shift (cm^{-1})	ε (M^{-1}cm^{-1})	Φ_F ^{b} (%)
**Toluen**	33.9	522,551	591	2237	17400	39.8
**THF**	37.4	555	608	1571	11900	36.5
**EA**	38.1	546	598	1593	9400	38.8
**DCM**	40.7	553	623	2032	14400	42.9
**DMSO**	45.1	578	648	1869	18900	4.4

^{a}**E_T(30)** is the solvent polarity parameter[1].

^{b}**Φ_F** is the absolute fluorescence quantum yield with an integrating sphere.

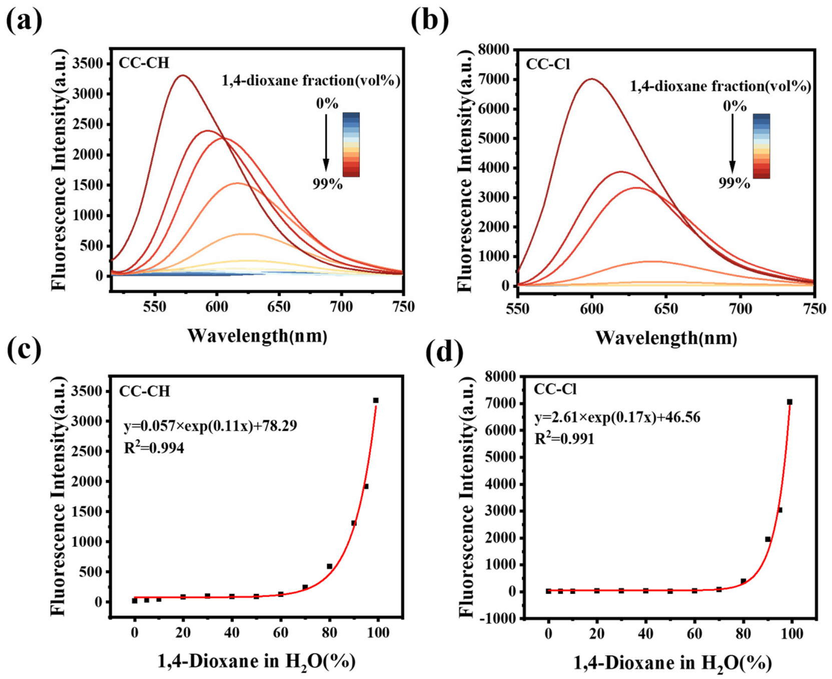

Figure S1. Fluorescence spectra of (a) CC-CH and (b) CC-Cl in H_2O/1,4-dioxane mixtures with different 1,4-dioxane fractions. Plots of fluorescence intensity of (c) CC-CH and (d) CC-Cl in H_2O/1,4-dioxane mixtures *vs* different 1,4-dioxane fractions. CC-CH: λ_{ex}= 492 nm, CC-Cl: λ_{ex}= 522 nm.

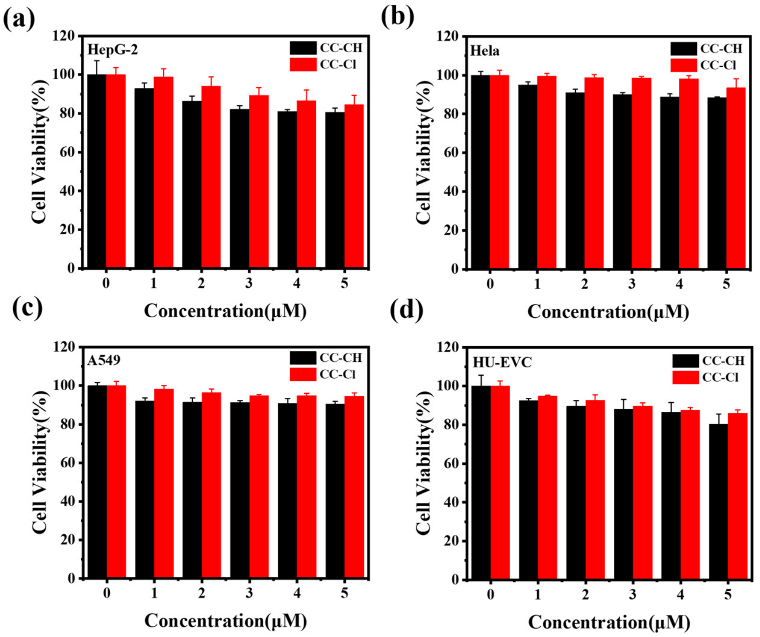

Figure S2. Cytotoxicity of CC-CH and CC-Cl in (a) HepG-2, (b) Hela, (c) A549, and (d) HU-EVC cells.

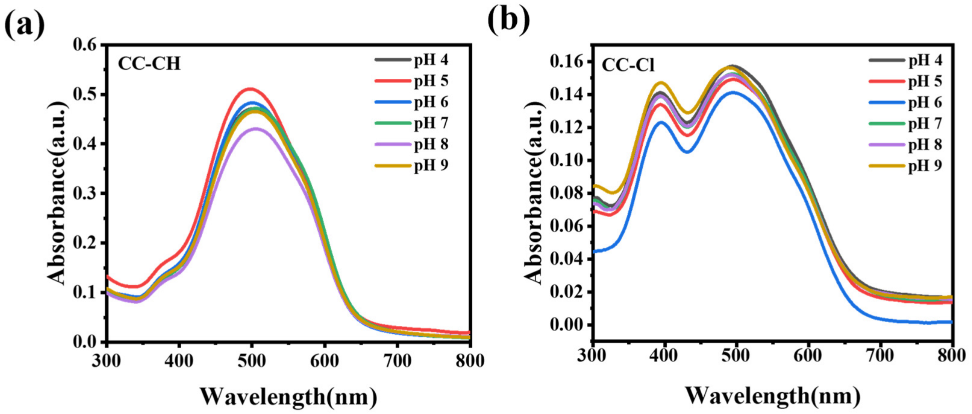

Figure S3. The absorption spectra of (a) CC-CH and (b) CC-Cl in PBS with different pH values (pH 4-9).

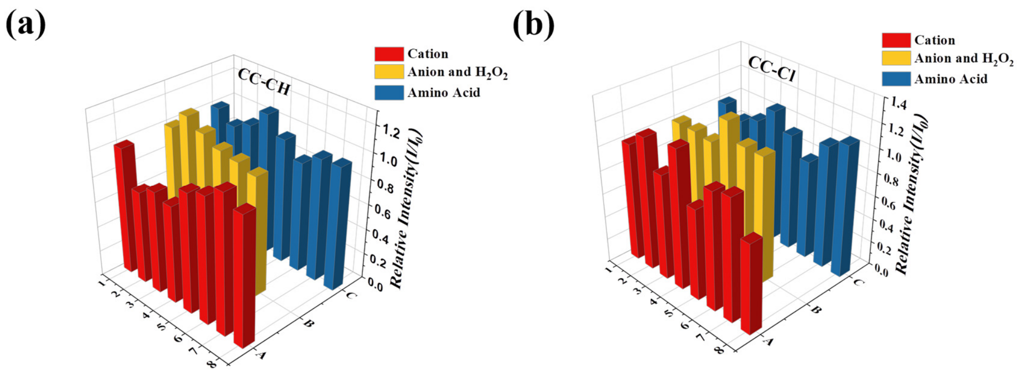

Figure S4. The relative fluorescence intensity (I/I_0) of (a) CC-CH and (b) CC-Cl to various interfering substances (100 μM) in PBS (pH =7.4).

A1/B1/C1, blank; A2, Fe^{3+}; A3, Mg^{2+}; A4, Ca^{2+}; A5, Cu^{2+}; A6, Mn^{2+}; A7, K^{+}; A8, Na^{+}; B2, SO_4^{2-}; B3, CO_3^{2-}; B4, HCO_3^{-}; B5, ClO^{-}; B6, H_2O_2; C2, Val; C3, Leu; C4, Pro; C5, Ser; C6, GSH; C7, Cys; C8, Hcy.

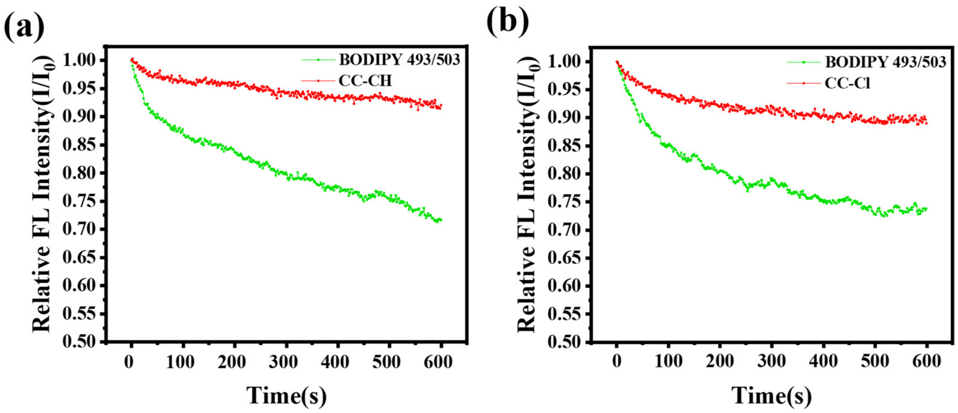

Figure S5. The relative fluorescence intensity (I/I_0) in HepG-2 cells stained with (a) CC-CH (2.5 μM) or (b) CC-Cl (2.5 μM) and BODIPY 493/503 (0.2 μM) upon continuous scanning by 488 nm laser (300 scans in ten minutes at two-second intervals).

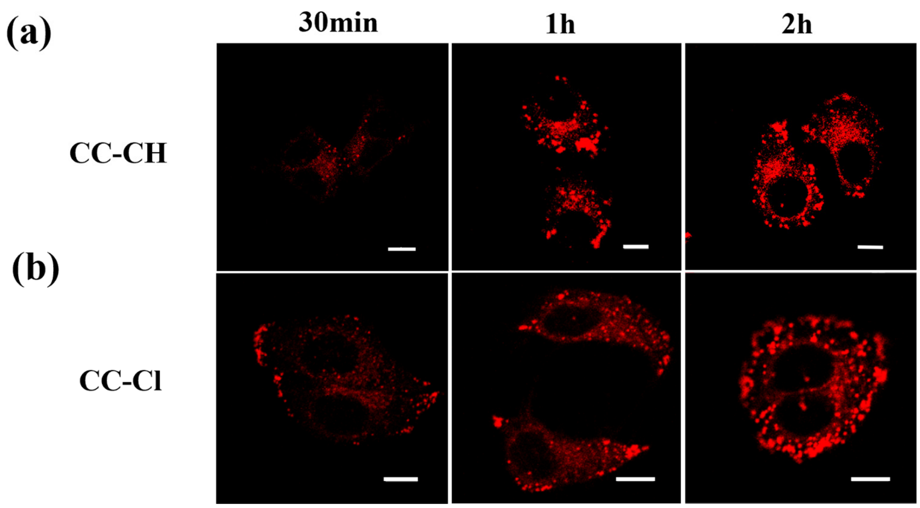

Figure S6. Confocal fluorescence images of (a) CC-CH (2.5 μM) and (b) CC-Cl (2.5 μM) in HepG-2 cells at different incubation times. Scale bar: 10 μm. Red channel: λ_{ex}= 488 nm, λ_{em} = 568-643 nm.

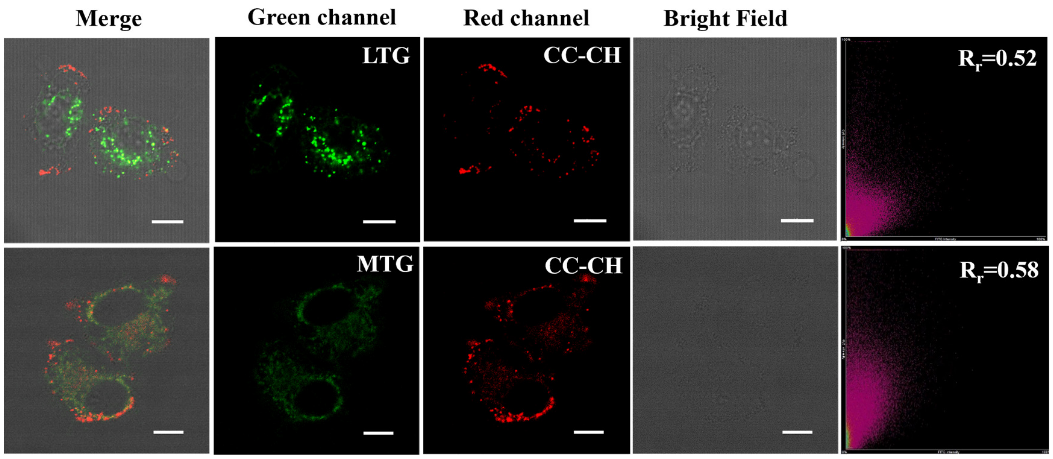

Figure S7. Colocalization images of CC-CH (2.5 μM) with LTG (0.2 μM) and MTG (0.2 μM) in HepG-2 cells. Scale bar: 10 μm. Green channel: λ_{ex}= 488 nm, λ_{em}= 500-530 nm. Red channel: λ_{ex}= 488 nm, λ_{em} = 568-643 nm.

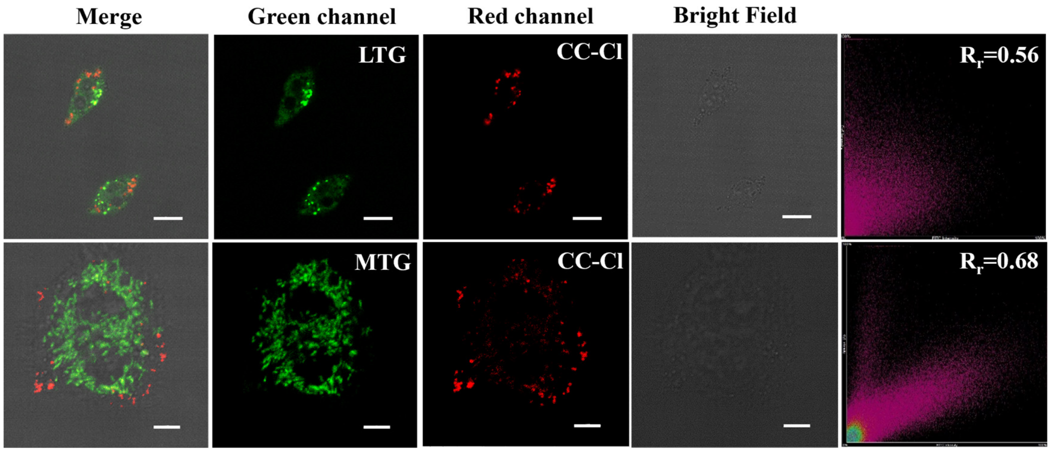

Figure S8. Colocalization images of CC-Cl (2.5 μM) with LTG (0.2 μM) and MTG (0.2 μM) in HepG-2 cells. Scale bar: 10 μm. Green channel: λ_{ex}= 488 nm, λ_{em}= 500-530 nm. Red channel: λ_{ex}= 488 nm, λ_{em} = 568-643 nm.

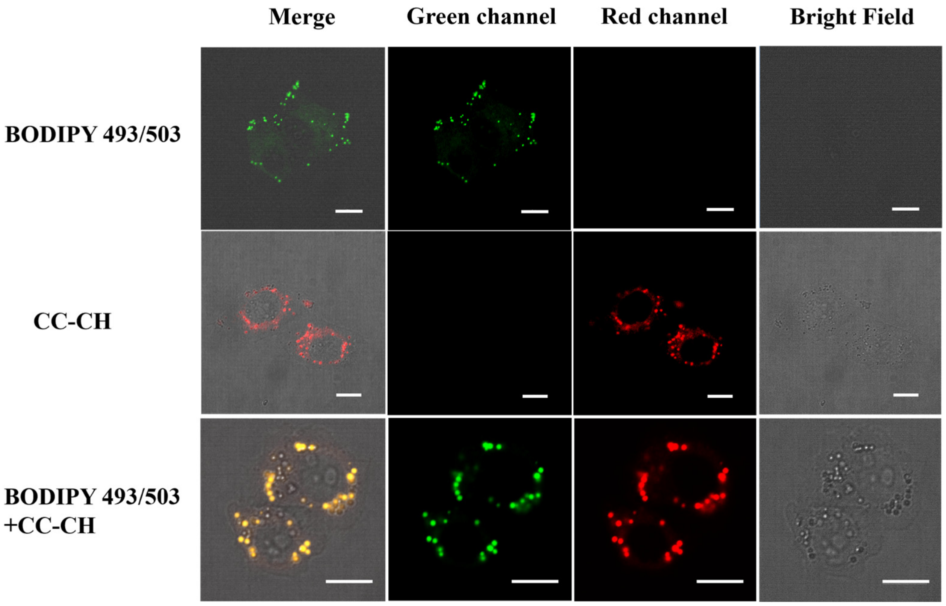

Figure S9. Confocal fluorescence images of BODIPY 493/503 (0.2 μM), CC-CH (2.5 μM), and BODIPY 493/503(0.2 μM) with CC-CH (2.5 μM) in HepG-2 cells. Scale bar: 10 μm. Green channel: λ_{ex}= 488 nm, λ_{em}= 500-530 nm. Red channel: λ_{ex}= 488 nm, λ_{em} = 568-643 nm.

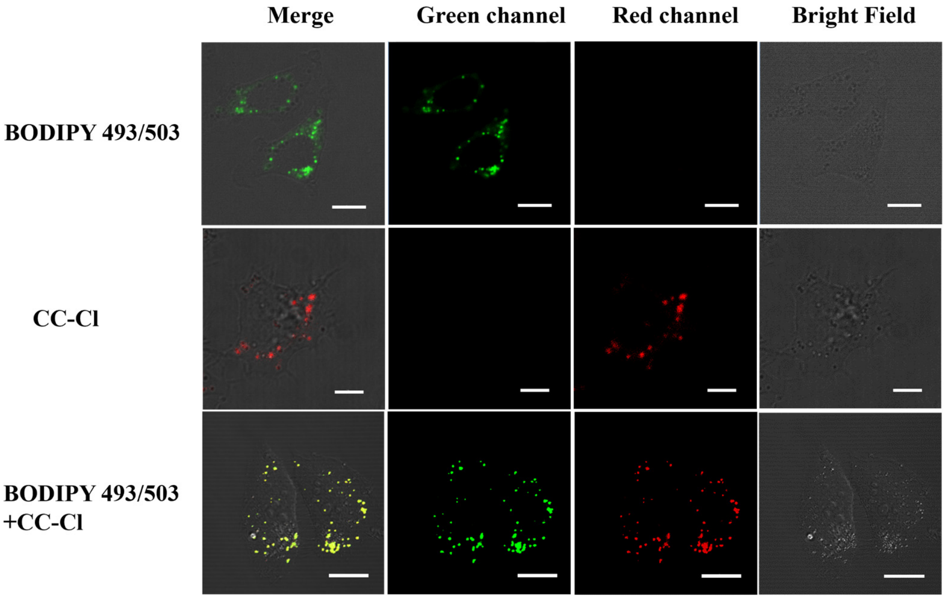

Figure S10. Confocal fluorescence images of BODIPY 493/503(0.2 μM), CC-Cl (2.5 μM), and BODIPY 493/503(0.2 μM) with CC-Cl (2.5 μM) in HepG-2 cells. Scale bar: 10 μm. Green channel: λ_{ex}= 488 nm, λ_{em}= 500-530 nm. Red channel: λ_{ex}= 488 nm, λ_{em} = 568-643 nm.

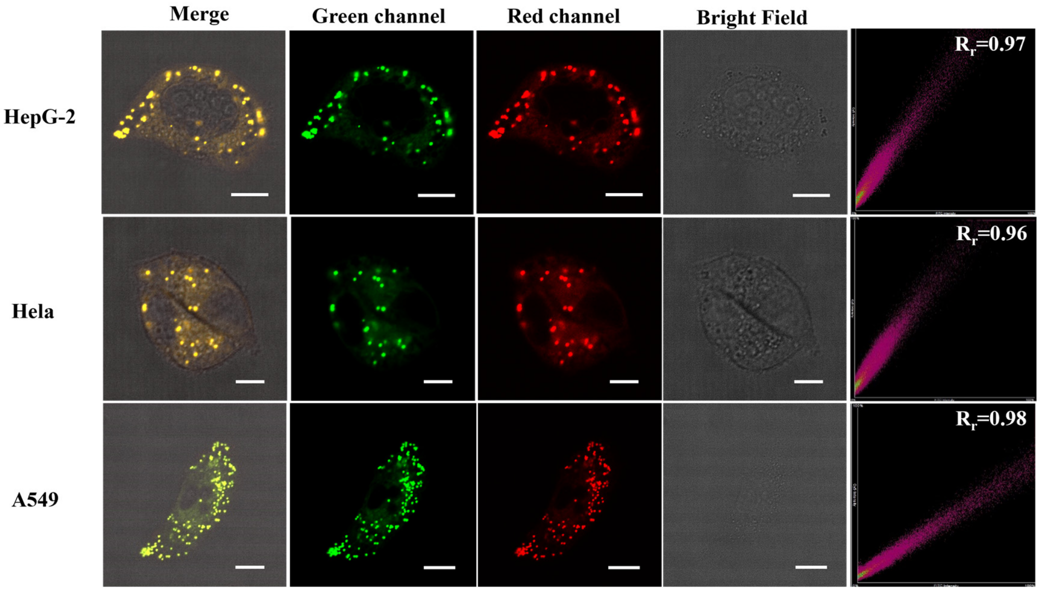

Figure S11. Colocalization images of CC-CH (2.5 μM) with BODIPY 493/503 in HepG-2, Hela, and A549 cells. Scale bar: 10 μm. Green channel: λ_{ex}= 488 nm, λ_{em}= 500-530 nm. Red channel: λ_{ex}= 488 nm, λ_{em} = 568-643 nm.

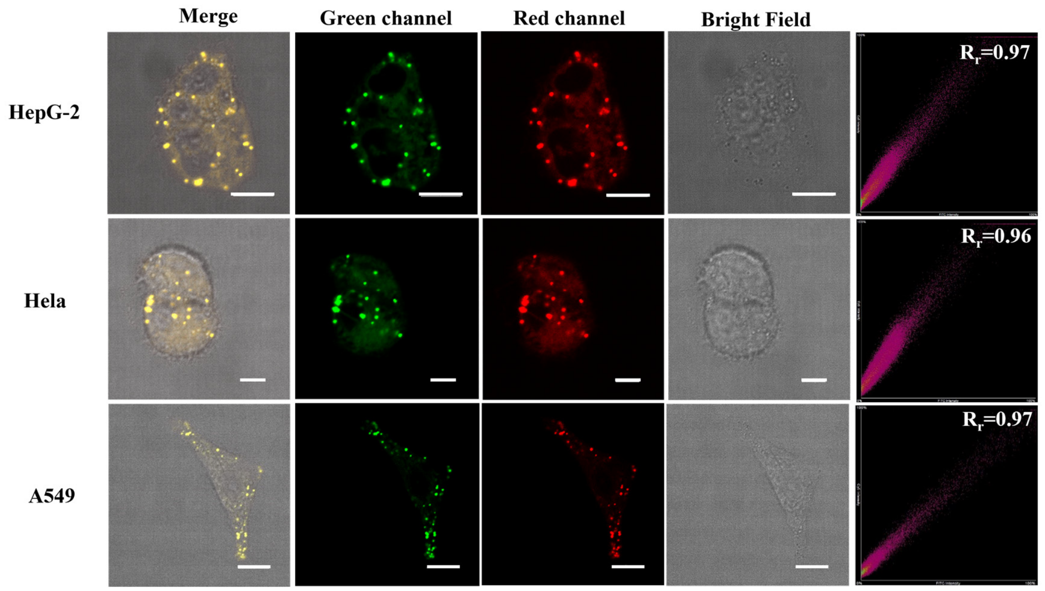

Figure S12. Colocalization images of CC-Cl (2.5 μM) with BODIPY 493/503 in HepG-2, Hela, and A549 cells. Scale bar: 10 μm. Green channel: λ_{ex}= 488 nm, λ_{em}= 500-530 nm. Red channel: λ_{ex}= 488 nm, λ_{em} = 568-643 nm.

\

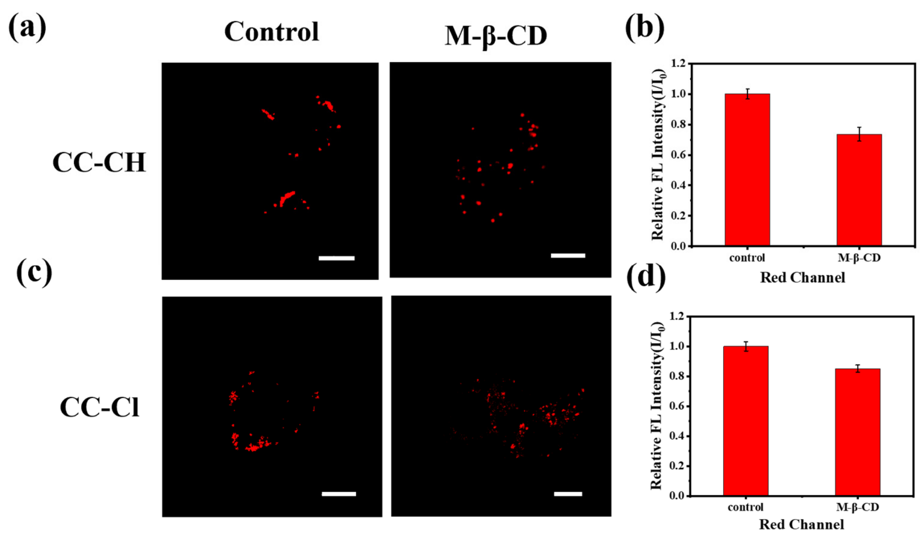

Figure S13. Confocal fluorescence images of HepG-2 cells treated with 2 mg/mL M-β-CD for 2h and then stained with (a) CC-CH (2.5μM) and (c) CC-Cl (2.5 μM) for 1h. The relative fluorescence intensity(I/I_0) of (b) CC-CH and

(d) CC-Cl of the control and M-β-CD-treated groups in the red channel. Scale bar: 10 μm. Red channel: λ_{ex}= 488 nm, λ_{em}= 568–643 nm.

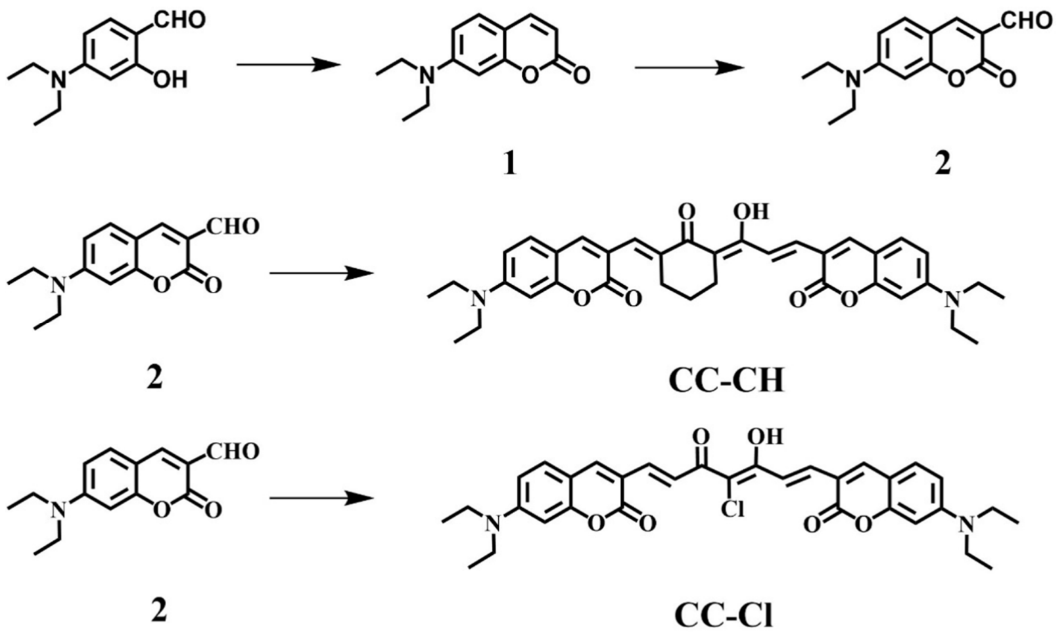

Scheme S1. Synthetic route to probes CC-CH and CC-Cl. Compounds 1-2 were synthesized according to previously reported procedures[2].

Synthesis of compound 7-(diethylamino)-2H-chromen-2-one (1):

2, 4-dihydroxybenzaldehyde (4.3g, 22.25 mmol) was dissolve in anhydrous ethanol (80 mL), diethyl malonate (7.5 mL, 50mmol) and piperidine (4 mL) were added. The reaction under reflux for 4h and cooled to room temperature. The solvent was evaporated in vacuo to obtain a reddish-brown viscous liquid. The reddish-brown viscous liquid was transferred to a 250 mL two-necked flask, concentrated hydrochloric acid (40 mL) and glacial acetic acid (40 mL) were added, and the mixture was heated to reflux for 10 h. After cooled to room temperature, the solution was transferred to ice water (150 mL). Aqueous NaOH solution was used to adjust to neutral pH, which was filtered with suction, and the solid was washed three times with ultrapure water and

ethanol to obtain khaki solid 1 (3.96g, 82%).^{1}H NMR (400 MHz, DMSO-d_6) δ 7.82 (d, *J* = 9.3 Hz, 1H), 7.42 (d, *J* = 8.8 Hz, 1H), 6.68 (d, *J* = 8.9 Hz, 1H), 6.51 (s, 1H), 5.99 (d, *J* = 9.3 Hz, 1H), 3.42 (q, *J* = 7.1 Hz, 4H), 1.12 (t, *J* = 7.1 Hz, 6H).

Synthesis of compound 7-(diethylamino)-2-oxo-2H-chromene-3-carbaldehyde (2):

Under a nitrogen atmosphere, 5 mL phosphorus oxychloride ($POCl_3$) was added to a two-necked flask, and then 7.2 mL anhydrous N, N-Dimethylformamide (DMF) was added dropwise to $POCl_3$. The temperature was raised to 50 °C and stirred for 0.5 h to produce a gold colored reactive intermediate. A solution of compound 1 (3.9g, 18mmol) in anhydrous DMF (20.0 mL) was added to reaction mixture and reacted at 60 °C for 12 h. The reaction solution was cooled to room temperature and added into ice water (150 mL). Aqueous NaOH solution was used to adjust to neutral pH, which was filtered with suction and washed the solid three times with ultrapure water and ethanol. Ethanol was used to obtain orange needle-like crystalline solid 2 (2.7 g, 69%). ^{1}H NMR (400 MHz, $CDCl_3$-*d*) δ 10.13 (s, 1H), 8.25 (s, 1H), 7.41 (d, *J* = 9.0 Hz, 1H), 6.64 (d, *J* = 9.0 Hz, 1H), 6.49 (s, 1H), 3.48 (q, *J* = 7.2 Hz, 4H), 1.25 (t, *J* = 7.1 Hz, 6H).

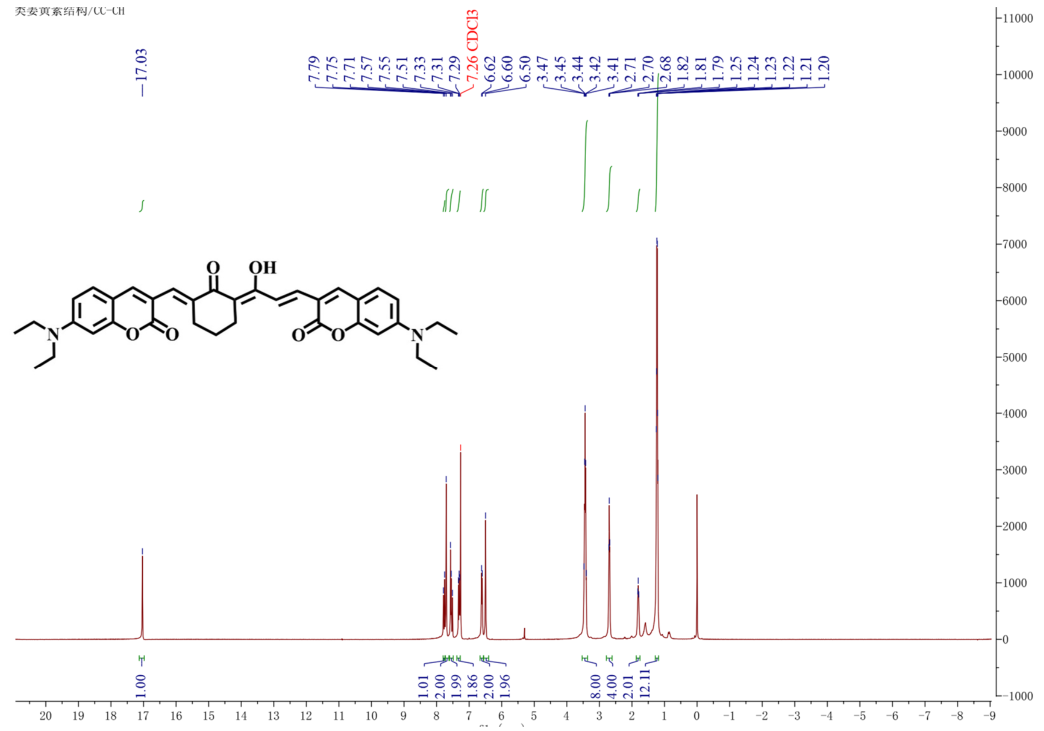

Figure S14. ^{1}H NMR of CC-CH.

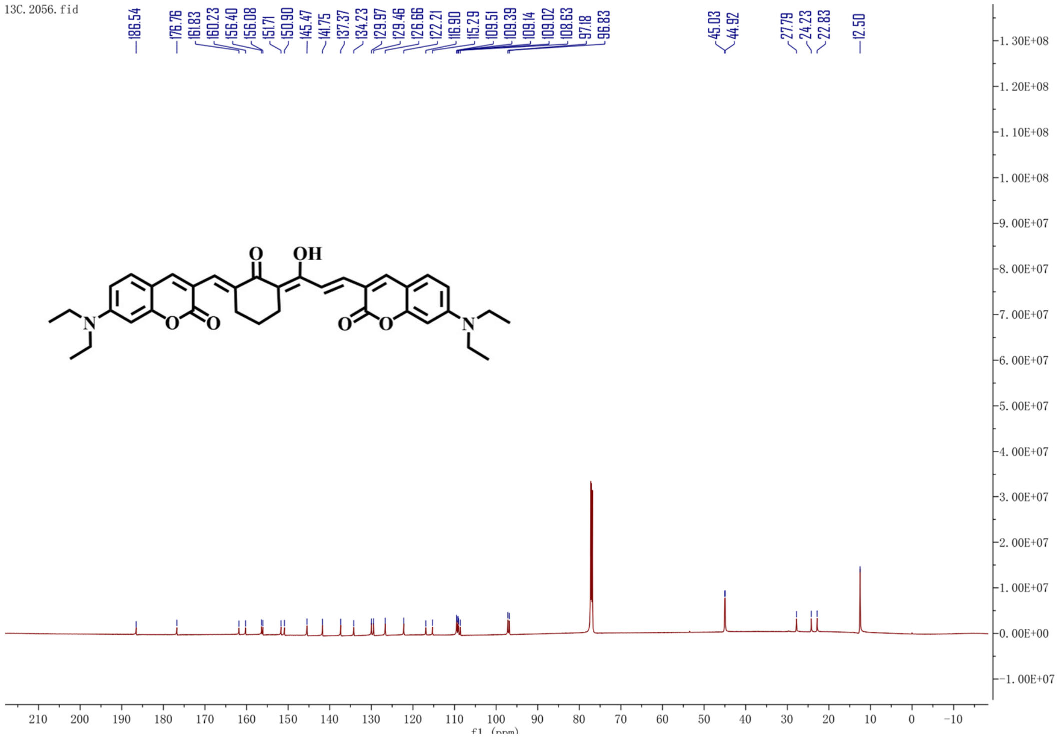

Figure S15. ^{13}C NMR of CC-CH.

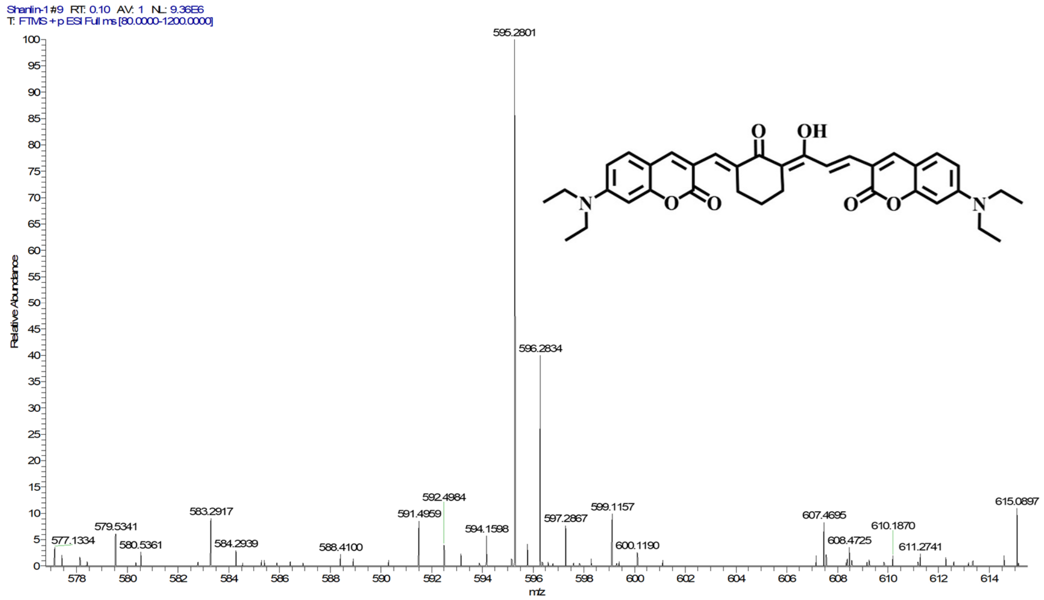

Figure S16. ESI-MS spectra of CC-CH.

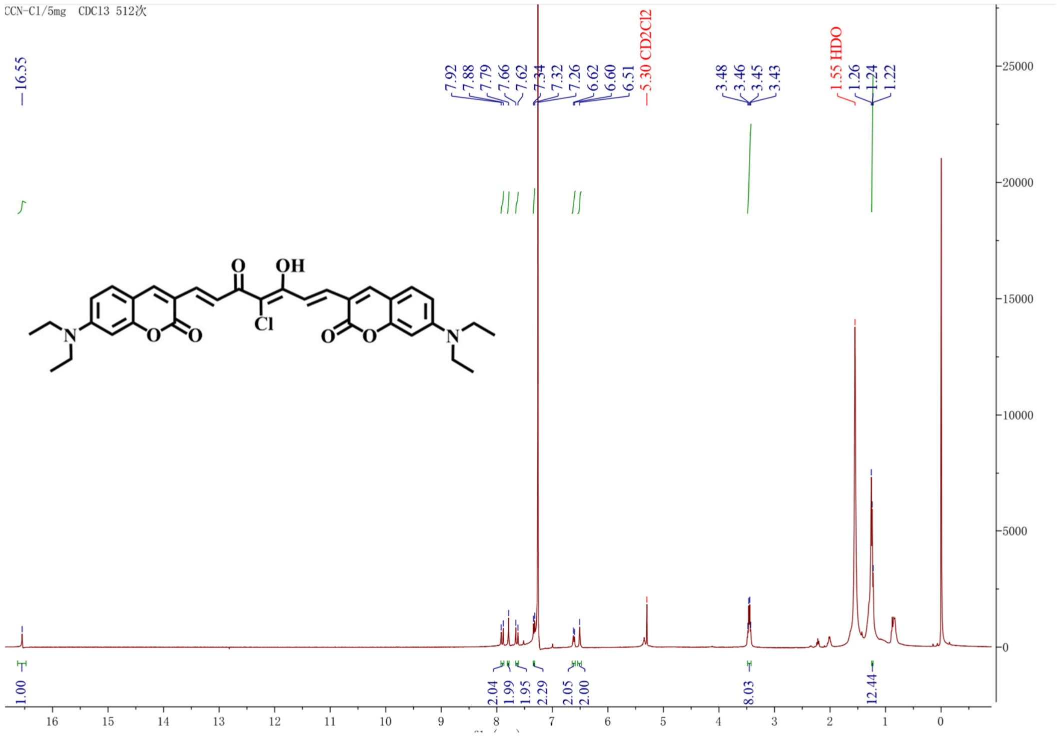

Figure S17. ^{1}H NMR of CC-Cl.

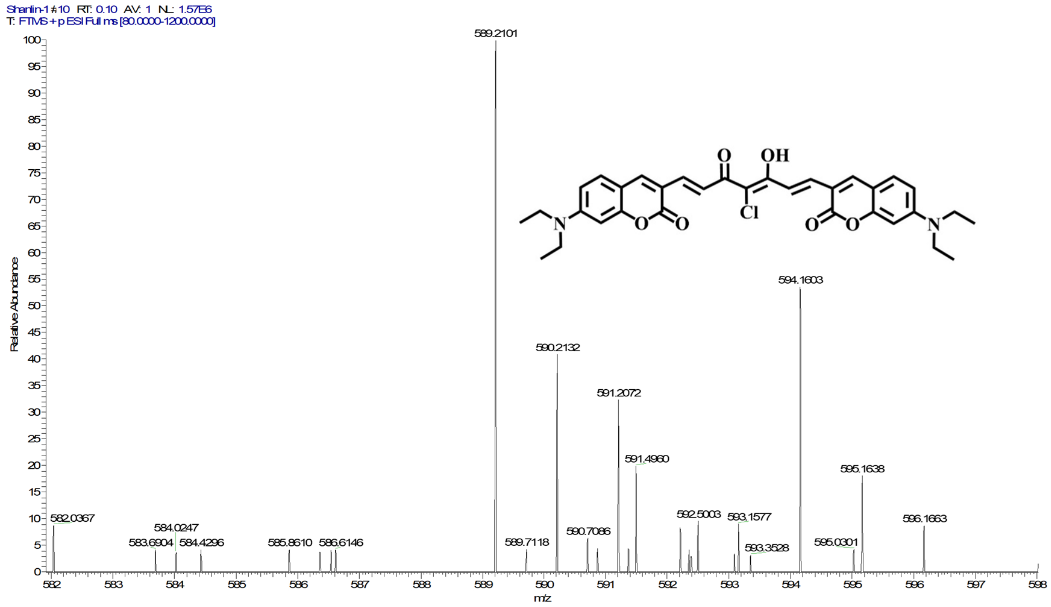

Figure S18. ESI-MS spectra of CC-Cl.

References

1. Reichardt, C. Solvatochromic Dyes as Solvent Polarity Indicators. *Chem. Rev.* **1994**, *94*, 2319-2358.
2. Zhang, Y.; Teng, H.; Gao, Y.; Afzal, M.W.; Tian, J.; Chen, X.; Tang, H.; James, T.D.; Guo, Y. A general strategy for selective detection of hypochlorous acid based on triazolopyridine formation. *Chin.Chem.Lett.* **2020**, *31*, 2917-2920, doi:10.1016/j.cclet.2020.03.020.
